# Influence of Different Ratios of *Lactobacillus delbrueckii* subsp. *bulgaricus* and *Streptococcus thermophilus* on Fermentation Characteristics of Yogurt

**DOI:** 10.3390/molecules28052123

**Published:** 2023-02-24

**Authors:** Tong Dan, Haimin Hu, Jiale Tian, Binbin He, Jiahui Tai, Yanyan He

**Affiliations:** 1 Key Laboratory of Dairy Biotechnology and Engineering, Ministry of Education, Department of Food Science and Engineering, Inner Mongolia Agricultural University, Hohhot 010018, China; 2 Key Laboratory of Dairy Products Processing, Ministry of Agriculture and Rural Affairs, Department of Food Science and Engineering, Inner Mongolia Agricultural University, Hohhot 010018, China; 3 Inner Mongolia Key Laboratory of Dairy Biotechnology and Engineering, Hohhot 010018, China

**Keywords:** starter culture, fermentation characteristics, volatile flavour compounds, SPME–GC–MS

## Abstract

Lactic acid bacteria (LAB) are industrially important bacteria that are widely used in the fermented food industry, especially in the manufacture of yogurt. The fermentation characteristics of LAB are an important factor affecting the physicochemical properties of yogurts. Here, different ratios of *L. delbrueckii* subsp. *bulgaricus* IMAU20312 and *S. thermophilus* IMAU80809 were compared with a commercial starter JD (control) for their effects on viable cell counts, pH values, titratable acidity (TA), viscosity and water holding capacity (WHC) of milk during fermentation. Sensory evaluation and flavour profiles were also determined at the end of fermentation. All samples had a viable cell count above 5.59 × 10^7^ CFU/mL at the end of fermentation, and a significant increase in TA and decrease in pH were observed. Viscosity, WHC and the sensory evaluation results of one treatment ratio (A3) were closer to the commercial starter control than the others. A total of 63 volatile flavour compounds and 10 odour-active (OAVs) compounds were detected in all treatment ratios and the control according to the results from solid-phase micro-extraction–gas chromatography–mass spectrometry (SPME–GC–MS). Principal components analysis (PCA) also indicated that the flavour characteristics of the A3 treatment ratio were closer to the control. These results help us understand how the fermentation characteristics of yogurts are affected by the ratio of *L. delbrueckii* subsp. *bulgaricus* to *S. thermophilus* in starter cultures; this is useful for the development of value-added fermented dairy products.

## 1. Introduction

The consumption of yogurt has increasing around the world; it is one of the most popular fermented dairy products due to its nutritional value, health benefits and sensory properties [1]. Chemically, yogurt is a complex gel system incorporating protein, polysaccharide and lipids in its structure. It is produced from milk by fermentation, one of the oldest methods practiced by human beings for the transformation of milk into products with an extended shelf life [2]. Yogurt is a balanced food supplying 0~3.5% fat, 5~6% protein, 4.6~5.2% lactose and minerals including calcium (Ca; 0.12~0.14%), phosphorus (P; 0.09~0.11%), sodium (Na), potassium (K), magnesium (Mg), iron (Fe), copper (Cu) and zinc (Zn). This food also provides consumers with several vitamins including vitamin A, B_6_, B_12_ and C amongst others [3]. Besides nutritional properties, yogurt provides a spectrum of interacting microorganisms that are considered beneficial for human gut health [4]. Typical characteristics of yogurt include its smooth viscous texture, sharp taste and unique flavour, all of which make it an attractive food ingredient [2,5]. Yogurt is produced from milk fermented with co-cultures of *L. delbrueckii* subsp. *bulgaricus* and *Streptococcus thermophiles*, *Lactococcus lactis* or *Lactobacillus plantarum.* These species are commonly known as ‘yogurt starter cultures’ [6]. Strains of *L. delbrueckii* subsp. *bulgaricus* and *S. thermophilus* have been isolated from a variety of habitats, particularly traditionally fermented food [7]. These strains are defined as a ubiquitous family of microbes that can ferment glucose into lactic acid as the major catabolic end product in a specific dairy environment.

Starter cultures are one of the most important factors in the delivery of the technological and organoleptic characteristics of fermented dairy food [8]. The ratio of *L. delbrueckii* subsp. *bulgaricus* and *S. thermophilus* in yogurt starter cultures has attracted wide attention. Generally, the ratio of *L. delbrueckii* subsp. *bulgaricus* and *S. thermophilus* in yogurt starter cultures is 1:1 or 1:2. The different ratios of starter cultures have a significant effect on fermentation time, pH, viscosity and other characteristics of the product. Alline et al. concluded that a long fermentation time reduced the efficiency of yogurt production and increased production costs and the rate of contamination by harmful microorganisms [9]. Dan et al. found that different ratios of *L. delbrueckii* subsp. *bulgaricus* and *S. thermophilus* had different viable cell counts [10]. Under the same fermentation conditions, the selection of starter cultures with good fermentation characteristics can improve the texture of yogurt. *L. delbrueckii* subsp. *bulgaricus* and *S. thermophilus* used in a mixed culture have a symbiotic relationship in milk when used as a yogurt starter culture via the exchange of metabolites that are necessary for the growth of each bacterium [11,12]. For example, *S. thermophilus* uses the peptides and free amino acids produced by *L. delbrueckii* subsp. *bulgaricus*, while *L. delbrueckii* subsp. *bulgaricus* uses the pyruvic acid, formic acid, folic acid and long-chain fatty acids produced by *S. thermophilus* [13]. Mixed fermentation with the two microorganisms gives the yogurt good textural properties. In addition, the production of metabolites such as amino acids and short peptides contributes to the formation of flavour substances in the yogurt [14].

Our enjoyment of yogurt is governed by our perception of aroma, taste and texture. Amongst these, flavour has the greatest effect on consumer acceptance and preference. The fermentation of lactose produces lactic acid, which denatures milk proteins and thus gives yogurt its texture (by forming a coagulated gel) and its characteristic flavour. A small number of by-products are also produced during this process. These by-products give yogurt its specific aroma and flavour [2]. The lactic acid and diverse volatile flavour compounds that impart the distinctive flavour of yogurt are produced with *L. delbrueckii* subsp. *bulgaricus* and *S. thermophilus.* To date, a large number of volatile flavour compounds have been found in yogurt [15]. For example, acetic acid gives fermented milk a sharp, pungent odour, acetaldehyde in certain concentrations gives yogurt a refreshing aromatic flavour and formic acid ethenyl ester has a fruity and floral flavour that greatly reduces the bitterness of fatty acids and amines [16]. Some investigations have shown that different ratios of *L. delbrueckii* subsp. *bulgaricus* and *S. thermophilus* in starter cultures have a critical influence on the resulting aroma of yogurt and are important for determining the overall flavour of the final product [10].

In previous studies, some strains such as *L. delbrueckii* subsp. *bulgaricus* IMAU20312 and *S. thermophilus* IMAU80809 were identified as having excellent fermentation properties, e.g., lactic acid production, viscosity, syneresis [17,18]. The main objectives of this study were to determine the effect of different ratios of *L. delbrueckii* subsp. *bulgaricus* IMAU20312 and *S. thermophilus* IMAU80809 on viable cell counts, pH, titratable acidity (TA), viscosity and water holding capacity (WHC) during fermentation, alongside sensory evaluation and flavour profiles made at the end of the fermentation, and to compare these with a commercial starter. This study reported on the effects of different ratios of *L. delbrueckii* subsp. *bulgaricus* and *S. thermophilus* as compound starter cultures on fermentation characteristics (such as acidity, viscosity and water-holding capacity) and flavour production during milk fermentation. Chemometrics were determined using heatmap analysis and principal components analysis (PCA) on screen characteristic volatiles of different fermented milks, with the aim of providing a reference for the development and use of starter cultures. This is important for understanding the relationship between *L. delbrueckii* subsp. *bulgaricus* and *S. thermophilus* and their relative contributions to the fermentation process.

## 2. Results

### 2.1. Growth of Starter Cultures during Fermentation

Fermentation time is an important indicator for identifying the best lactic acid bacteria for use as the starter cultures. Changes in fermentation time and viable cell counts of the reference JD (control) and different combinations of the starter cultures during fermentation are shown in Table 1. The fermentation times of all five groups of compound starter cultures were close to the control and had excellent acid production characteristics. In addition, the viable cell counts of all samples and the control increased rapidly and reached the maximum count during fermentation. The viable bacterial count of the samples reached 5.73 × 10^7^ (A1), 5.75 × 10^7^ (A2), 5.68 × 10^7^ (A3), 5.69 × 10^7^ (A4) and 5.59 × 10^7^ (A5) CFU/mL at the end of fermentation, respectively, which were all close to the control (5.70 × 10^7^ CFU/mL). There were no significant differences between the control and the different treatments.

### 2.2. Changes in pH and TA during Fermentation

**Figure 1:**
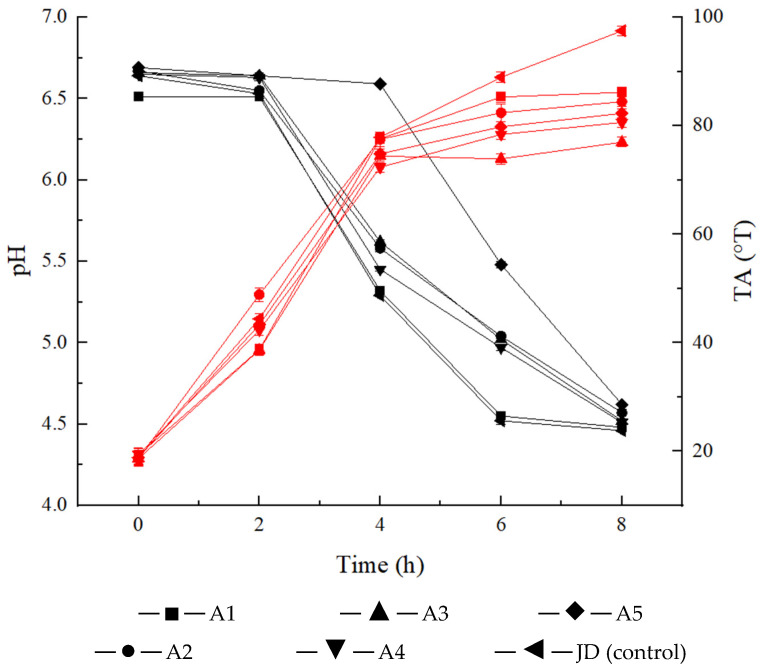
Changes in pH and TA during fermentation. The black and red lines represent pH and TA of the samples, respectively.

The changes in pH and TA during fermentation are shown in Figure 1. The pH and TA of the fermented milk in the five groups of mixed starter cultures of *L. delbrueckii* subsp. *bulgaricus* IMAU20312 and *S. thermophilus* IMAU80809 and the control strain showed opposite trends. At the beginning of fermentation, the pH of all samples was 6.51 (A1), 6.57 (A2), 6.66 (A3), 6.65 (A4) and 6.69 (A5), and the TA was 19.3 (A1), 18.6 (A2), 18.65 (A3), 19.4 (A4) and 18.85 (A5) °T, which was not significantly different from the control (*p* > 0.05). The TA of the samples continued to rise and stayed below 100 °T throughout fermentation. At the end of fermentation, the highest TA (86.15 °T) was achieved in the A1 sample, but it was still lower than that of the control (97.5 °T).

### 2.3. Viscosity and WHC during Fermentation

Changes in the viscosity and WHC of all samples and the control during fermentation are shown in Table 2. At the end of fermentation, the viscosities of the yogurt in all samples and the control were 580 (A1), 590 (A2), 615 (A3), 560 (A4), 580 (A5) and 590 (control) mPa·s. There were significant differences in the viscosity of the samples, indicating that the ratio of *L. delbrueckii* subsp. *bulgaricus* IMAU20312 and *S. thermophilus* IMAU80809 in the starter culture had a clear influence on the viscosity of the yogurt.

The WHC of all samples and the control had significantly decreased by the end of fermentation (*p* < 0.05). Higher WHC values indicated a strong tissue state and improved retention of aromas. At the end of fermentation, the WHC was highest in the A1, A2 and A3 samples, and lowest in the A4 sample (Table 2). These results confirmed that the ratio of *L. delbrueckii* subsp. *bulgaricus* IMAU20312 and *S. thermophilus* IMAU80809 in the starter culture affected yogurt consistency.

### 2.4. Analysis of Volatile Flavour Compounds in Yogurt

#### 2.4.1. Analysis of Volatile Flavour Compounds

Fermented dairy products share a common sour or tart taste. However, they each have unique flavours that enable them to be distinguished from each other. The volatile flavour compounds detected in all samples at the end of the fermentation using SPME–GC–MS are shown in Table S1. A total of 63 volatile flavour compounds were identified using the scan method and the standard spectrum library of NIST in all samples (A1–A5) and the commercial starter culture. The volatile flavour compounds were grouped by their chemical family as carboxylic acids, aldehydes, ketones, alcohols, esters and aromatic hydrocarbons.

Carboxylic acids are very important for conferring strong odour notes to dairy products [19]. Nineteen carboxylic acids were identified in all samples and the control. Acetic acid is the main product of fermentation, indicative of glucose consumption/breakdown by the starter cultures [20]. At the end of fermentation, acetic acid concentrations of 1.8, 0.57 and 0.17 µg/L were achieved in the A1, A2 and control treatments (Table S1). Acetic acid is the most important acid in yogurt and imparts strong, pungent, vinegary and acidic odours [21]. Hexanoic acid is also produced; it increases the yogurt aroma and significantly contributes to the ‘rancid, cheese-like’ odour and flavour [22]. High levels of hexanoic acid were detected in all samples and the control; in particular, a final concentration of 12.10 µg/L was detected in the A4 samples, which was higher than the control (1.69 µg/L). Octanoic acid can confer a ‘fruit, caramel’ flavour to yogurt [23]. As with hexanoic acid, high concentrations of octanoic acid were detected in the A4 samples.

Aldehyde compounds have a low taste threshold, and even trace concentrations play an important role in the flavour of dairy products [10]. Acetaldehyde is recognized as an important flavour component, endowing a green apple or nutty flavour to yogurt [24]. Seven aldehyde compounds were detected in the volatile fraction of all samples and the control. High levels of acetaldehyde were detected in the A1, A3 and A4 samples at concentrations of 5.94, 2.79 and 22.33 µg/L, respectively. The branched-chain aldehyde, 3-methylbutanal, is produced from isoleucine and leucine catabolism [25] and was detected in the A1, A4 and A5 samples at concentrations of 0.96, 0.96 and 1.02 µg/L, respectively. Benzaldehyde may be produced from phenylacetaldehyde or cinnamic acid through α-oxidation or β-oxidation [26] and was detected in all samples and the control; it is worth mentioning that the highest concentration of 2.40 µg/L was found in the A1 samples.

In total, eleven ketone compounds were identified in all samples and the control. 2,3-butanedione and acetoin are volatile products of citrate metabolism, endowing buttery and creamy flavours to yogurt [27]; 2,3-butanedione was detected in samples A2 and A3, and at particularly high levels (4.72 µg/L) in the A2 sample. Acetoin was reduced from 2,3-butanedione and decarboxylated by α-acetolactic acid [28], and was detected in all samples at concentrations of 4.36, 16.59, 2.65, 21.51 and 7.86 µg/L, respectively. With the exception of the A3 sample, the concentration of acetoin in all samples was higher than in the control. 2-nonanone was also detected in all samples and the control. It is an important volatile compound that imparts a ‘buttery, creamy, vanilla’ flavour to yogurt [10].

Alcohol compounds are formed through lactose fermentation, amino acid metabolism, methyl ketones or aldehyde reduction [29]; seven alcohol compounds were identified. Amongst these, 1-nonanol is an important volatile flavour compound with a fatty odour that contributes to dairy product flavour [18]. It was detected in the A4 and A5 samples and the control at concentrations of 3.25, 1.97 and 0.06 µg/L, respectively.

Ester compounds can be formed from esterification reactions, which occur via lactose fermentation or amino acid degradation [30]. Three ester compounds were identified. Amongst these, formic acid ethenyl ester and hexanoic acid ethyl ester are common flavour compounds that contribute to a good flavour in fermented dairy products. Formic acid ethenyl ester was only detected in the A3 samples at a concentration of 12.66 µg/L. Hexanoic acid ethyl ester was detected in all samples at a range of 0.09~0.69 µg/L.

Aromatic hydrocarbons are often used as a substrate to promote the growth of microorganisms [31]. Fifteen aromatic hydrocarbons were detected. Among these, some were at high concentrations, e.g., ethylene oxide, 1-nonyne. However, these compounds are considered to have little effect on fermented dairy products due to their high threshold value [15].

#### 2.4.2. PCA

**Figure 2:**
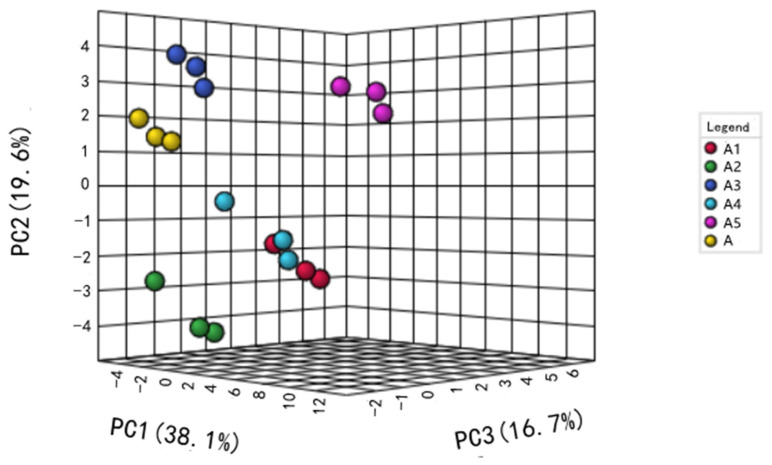
PCA plot of volatile flavour profiles at the end of fermentation. Five different ratios of *L. delbrueckii* subsp. *bulgaricus* IMAU20312 and *S. thermophilus* IMAU80809 were used as mixed starter cultures: A1 = 1:1, A2 = 1:10, A3 = 1:100, A4 = 1:1000, A5 = 1:2000, A = JD (commercial yogurt starter culture as a control).

PCA is a multivariate analysis technique that extracts important information from data, represents it as a new set of orthogonal variables called principal components and displays the patterns of similarity amongst observations and variables as points on a graph [32]. The PCA model had a high R^2^Y value of 0.997 and Q^2^ value of 0.994, respectively, indicating that the PCA model was accurate and provided good predictability. A total of 38.1%, 19.6% and 16.7% of the variability for PC1, PC2 and PC3 was accounted for. Three groups were separated clearly on the PCA plot (Figure 2). The first group included the A3 and control samples. The second group contained the A1 and A2 samples, and the third group contained the A4 and A5 samples.

### 2.5. Heatmap

**Figure 3:**
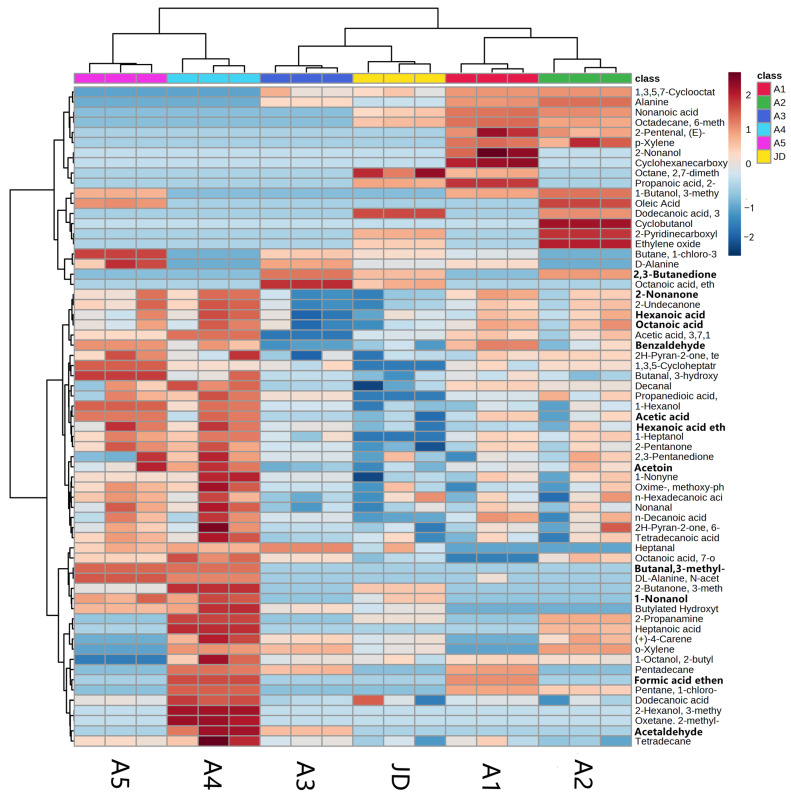
Heatmap of volatile flavour compounds in all samples and the control. The metric of the x-axis is five different ratios of *L. delbrueckii* subsp. *bulgaricus* IMAU20312 and *S. thermophilus* IMAU80809 in starter cultures and the commercial starter culture control, and the y-axis is the concentration of volatile flavour compounds in all samples and the control (JD).

To analyse the clustering of the volatile flavour compounds in all samples and the control, a heatmap was produced based on intensity patterns (Figure 3). In the heatmap, the overall profile of the identified volatile flavour compounds were presented for all samples and the control. The colour intensity had a scale from dark red (maximum 2) to dark blue (minimum −2), and indicated the quantity of the volatile flavour compounds from high to low. All of the volatile flavour compounds produced were clustered into two groups: A4 and A5 were gathered into one group; and A1, A2, A3 and the control were in the other group. The A3 sample was closest to the control. This result was consistent with the PCA.

### 2.6. Sensory Evaluation

Sensory evaluation is an important indicator of whether a yogurt product will achieve consumer acceptance and global popularity [33]. The results for the sensory evaluation of colour, taste and flavour, and the texture of all samples and the control are shown in Table 3. The scores for the A3 samples were higher than the other four samples and closer to the commercial control. This result was in accordance with the PCA results, i.e., A3 had better characteristics than the other samples and could be considered as being close to the commercial control.

## 3. Discussion

Milk can be transformed into other food products, such as yogurt, using fermentation, which requires particular attributes of lactic acid bacteria [34]. The most effective strains are adapted to environmental conditions, produce acid rapidly from the lactose in milk and large amounts of volatile flavour compounds [2], such as diacetyl and acetaldehyde. Traditionally fermented dairy products rarely achieve fermentation as a result of a single bacterial strain and rely on spontaneous growth of bacteria from the environment. Usually, yogurt starter cultures are comprised of *L. delbrueckii* subsp. *bulgaricus* and *S. thermophilus* in a 1:1 or 1:2 ratio, but with the increasing demand for commercial starter cultures in the fermentation industry, the impact of using different ratios of *L. delbrueckii* subsp. *bulgaricus* and *S. thermophilus* on dairy flavour substances has been of increasing interest to the industry. However, little information is available on the effect of different combinations of *L. delbrueckii* subsp. *bulgaricus* and *S. thermophilus* on the flavour of yogurt produced by conventional milk fermentation. Today, starter cultures are carefully selected to produce dairy products with particular attributes. With the rapid development of the dairy industry, some tailor-made mixed cultures of known species of lactic acid bacteria have been developed as starter cultures. Mixed cultures promote biosynthesis of metabolites, which are important fermentation characteristics in a starter culture. These include mixtures with different proportions of *L. delbrueckii* subsp. *bulgaricus* and *S. thermophilus* with good fermentation characteristics that produce specific flavours. The main quality characteristics of yogurt include texture, taste, aroma and flavour [35]. In this study, the effect of five different combinations of *L. delbrueckii* subsp. *bulgaricus* and *S. thermophilus* on the fermentation characteristics of milk and on the volatile flavour compounds produced was evaluated. In addition, it should be noted that this study focused mainly on determining the differences in fermentation characteristics and flavour profiles between the different combinations and the commercial yogurt starter culture used as a control.

In this study, we evaluated the effect of different ratios of *L. delbrueckii* subsp. *bulgaricus* and *S. thermophilus* as starter cultures on pH, TA, viscosity, WHC and viable cell counts during the fermentation of yogurt. The fermentation characteristics of lactic acid bacteria are important factors in screening starter culture strains. One of the most important indicators for screening starter cultures is fermentation time. We observed different fermentation times in each sample compared with the control (Table 1). Donkor et al. suggested that yogurt tasted better when the titratable acidity of the fermented milk was controlled between 70 and 110 °T [36]. During fermentation, the TA increased significantly, and pH decreased in all samples and the control. At the end of fermentation, the TA was above 70 °T in all samples (Figure 1). As an indicator of viability, viable cell counts are considered a key parameter in the development of fermented dairy products. It is generally accepted that the viable cell counts in yogurt should be maintained at 10^7^ CFU/mL or higher [37]. In our study, the viable cell counts in all samples and the control were in the range of 5.59–5.75 × 10^7^ CFU/mL at the end of fermentation. The WHC of fermented dairy products is determined by the ability of three-dimensional reticulation of the proteins to retain water [38], probably due to the continuous increase in acid production and WHC of the strain during fermentation. The viscosity of all samples and the control (JD) increased significantly during fermentation, especially in the A3 sample, which reached 615 mPa·s, which was higher than the control group (590 mPa·s). Similarly, the WHC of A3 (57%) was higher than the control (47%) and the difference was significant at the end of fermentation.

Flavour is one of the most important characteristics of a food product and is an important determinant of consumer acceptability and preference. The sensory properties of dairy products depend to a large extent on the relative balance of flavour compounds in the milk derived from fat, protein or carbohydrates [39]. The distinctive flavour of yogurt is contributed by lactic acid and a complex mixture of aromatic compounds, including volatiles already present in the milk and particular compounds produced during milk fermentation [40]. GC–MS is the most popular technique used for fragrance analysis because of its ability to detect and quantify known compounds, identify unknown compounds and elucidate the chemical properties of molecules [41]. Hussein analysed the volatile components of fresh and fermented camels’ milk (*L. delbrueckii* subsp. *bulgaricus* and *S. thermophilus*) using GC–MS, and showed that lactic acid bacteria played an important role in the fermentation of this milk [41]. In our study, a total of 63 volatile flavour compounds were detected from the samples using GC–MS. To determine how each volatile compound contributed to the flavour profile of all samples, OAV values were calculated (Table 4). Generally, volatile compounds with OAVs ≥ 1 are considered sufficient to contribute to the flavour profile of the yogurt. In our study, some volatile compounds with OAVs > 1 were found at the end of fermentation, including acetaldehyde (OAV = 2.57), nonanal (OAV = 2.02), 2-nonanone (OAV = 1.99) and formic acid ethenyl ester (OAV = 1.41). Some volatile compounds were present at levels lower than the threshold value (0.1 ≤ OAV < 1, Table 4), but might still modify the overall flavour of the samples. There were significant differences in the quantities of these volatile compounds (OAVs ≥ 0.1) between the treatments with different ratios of *L. delbrueckii* subsp. *bulgaricus* and *S. thermophilus* in the starter cultures and the control. Thus, the ratio of *L. delbrueckii* subsp. *bulgaricus* and *S. thermophilus* in the starter cultures might be an important factor affecting the aroma of yogurt.

Chemometric techniques are extremely important and widely used for solving flavour chemistry problems. Amongst these, heatmaps and PCA are commonly used to interpret chromatographic data involving a large number of compounds and a large number of variables. Heatmaps are a clustering technique that complement PCA by grouping data sets according to their similarity or dissimilarity [47]. For example, Zhang et al. used heatmap analysis and PCA to characterise the volatile profiles of milk fermented using different starter cultures [48]. We evaluated the relationship between volatile aroma compounds in different samples using PCA and heatmaps and found that the composition of the volatile aroma compounds in the A3 sample was the closest to that of the commercial control group.

Sensory evaluation is a powerful tool that can aid communication amongst consumers and producer groups in the fermented dairy industry. Although the flavour characteristics of products are influenced by some key volatile flavour compounds, the final quality of the products is determined by texture attributes [49]. In order to confirm the effects of these compounds on the sensory attributes and general taste for consumers, sensory evaluations were made. In our study, the results of the sensory evaluation of five ratios of *L. delbrueckii* subsp. *bulgaricus* and *S. thermophilus* in the starter cultures and the control showed that the A3 sample had significantly better characteristics than the other combinations and was close to the control (Table 3). This result was consistent with the PCA and heatmap (Figure 2 and Figure 3). In addition, fermentation characteristics such as pH, TA, viscosity and WHC were important in determining consumer acceptance [50].

## 4. Material and Methods

### 4.1. Isolates, Growth Media and Inoculation Cultures

The bacterial strains *L. delbrueckii* subsp. *bulgaricus* IMAU20312 and *S. thermophiles* IMAU80809 were collected from traditional dairy products in Mongolia and the Gansu Province of China and were known to have good fermentation characteristics [51]. *L. delbrueckii* subsp. *bulgaricus* IMAU20312 and *S. thermophilus* IMAU80809 were activated in De Man, Rogosa and Sharpe (MRS) liquid medium (027312, Huankai Microbial, Guangdong, China) and M17 (HB0391, QuingDoa HopeBiol Co., Quingdau, China) liquid media at 37 °C for 24 h, respectively. The cells were collected using centrifugation at 5000× *g* for 5 min at 4 °C and re-suspended in phosphate-buffered saline (PBS; 0.8% NaCl, 0.02% KCl, 0.02% KH_2_PO_4_, 0.115% Na_2_HPO_4_; pH 7.4). Five proportional combinations of *L. delbrueckii* subsp. *bulgaricus* IMAU20312 and *S. thermophilus* IMAU80809 were used as mixed starter cultures and compared with a commercial yogurt starter culture JD (inoculation amount 0.03‰) supplied by Chr-Hansen (Horsholm, Denmark) as a reference control (Table 5).

### 4.2. Reagents

n-Alkane standards were obtained from AccuStandard, Inc. (New Haven, CT, USA). Whole milk powder was purchased from NZMP (Wellington, New Zealand). Skimmed milk powder was purchased from Auckland, New Zealand Heng Nature Co., Ltd. 1,2-Dichloro-benzene as an internal standard (ISTD) was purchased from Sigma–Aldrich (Steinheim, Germany).

### 4.3. Preparation of Yogurt Samples

Yogurts were prepared using a previously described method with some modifications [52]. Sterilized milk was prepared by reconstituting 11.5% (*w*/*v*) whole milk powder in distilled water and heating to 50 °C for 30 min. Sucrose was then added (to achieve 6.5%) when the temperature had increased to 60 °C. Two consecutive homogenization treatments (15 and 35 MPa) were completed at an inlet temperature of 65 °C after hydration for 30 min at 60 °C. The milk was heated at 95 °C for 5 min and then cooled to 42 °C (the incubation temperature) until it was used.

*L. delbrueckii* subsp. *bulgaricus* and *S. thermophilus* were mixed in the ratios 1:1 (A1), 1:10 (A2), 1:100 (A3), 1:1000 (A4) and 1:2000 (A5) and inoculated into milk; the inoculation quantity of *S. thermophilus* was 5 × 10^6^ CFU/mL in all combinations. Samples were incubated at 42 °C until the pH fell to approximately 4.5. All samples were then stored at −20°C until they were analysed using GC–MS.

### 4.4. Enumeration of Viable Cell Counts

At different times during fermentation (every 2 h), samples were diluted as appropriate and viable cells were enumerated as previously described in Meng et al. [53]. Specifically, all samples (1.0 mL) were diluted in 9 mL of sterile physiological saline (0.85%, *w*/*v*) and then appropriate dilutions made. Subsequently, each gradient dilution was plated on MRS agar medium. Plates were incubated under anaerobic conditions at 42 °C for 48 h. Colonies were counted on each plate that contained between 30 and 300 colonies and viable cell counts expressed as units (CFU)/mL. Means were determined from the results of replicate samples (*n* = 3).

### 4.5. Determination of pH and Titratable Acidity (TA)

Lactic acid is one of the main acids that contribute to the acidity of fermented milk. The pH was measured using a PHS-3C pH-meter (Leici Devices, Shanghai, China). The TA of samples was determined using the acid–base titration method as described in Bai et al. [54]. Specifically, 5 g yogurt were accurately weighed into tapered bottles and then 40 mL of distilled water added and titrated with 0.1 mol/L NaOH standard solution using 0.5% phenolphthalein as an indicator. TA was calculated using the following formula:

X = (c × V × 100)/(m × 0.1)(1)

where ‘X’ is the titrated acidity of the yogurt sample (°T), ‘c’ is the concentration of the NaOH standard solution (mol/L), ‘V’ is the volume of NaOH standard solution ‘consumed’ (mL), ‘m’ is the quantity of the yogurt sample (g).

### 4.6. Determination of Viscosity

The viscosity of 40 g samples of yogurt was measured using a BROOKFIELD DV-1 Viscometer (Shanghai, China) in a 50 mL centrifuge tube. All samples were stirred at 100 r/min for 30 s with a No. 4 spindle and a torque of 20%~100% [54].

### 4.7. Determination of Water Holding Capacity (WHC)

The WHC of yogurt was determined as described in Yao et al. [55]. Briefly, 15 g samples of yogurt were placed in funnels with filter paper and allowed to run through for 2 h. The filtrate was collected and weighed to determine the amount of water that had been excluded (% wt/wt) at room temperature. WHC was calculated using the following equation:

WHC (%) = 1 − (The quantity of filtrate (g)/The quantity of sample (g)) × 100%(2)

### 4.8. Solid-Phase Microextraction (SPME) Procedures

Volatile flavour compounds were collected using the headspace SPME technique [56]. Briefly, 50 µL of 1,2-dichlorobenzene as ISTD and 5 mL of sample were mixed in 20 mL glass vials (CNW Technologies, Germany) fitted with a PTFE/silicone septum. The final concentration of ISTD in each sample was 10 µg/L. All extractions were performed using SPME fibres (50/30 µm DVB/Carboxen/PDMS; Supelco, Inc., Bellefonte, PA, USA), the glass vials were kept at 50 °C for 60 min and the samples were stirred continuously with a magnetic stirring bar at 300 rpm. Subsequently, the fibre was immediately inserted into the injection port of a 7890B GC (Agilent Technologies, Inc., Palo Alto, CA, USA) for 5 min at 270 °C to desorb volatile compounds into the GC.

A 7890B gas chromatograph equipped with a 5977A mass selective detector (MSD; Agilent Technologies) was used to analyse the volatile flavour compounds of the samples. Details: column, HP-5MS column (30 m length, 0.25 mm inside diameter, 0.25 µm film thickness; California, USA, Agilent Technologies Inc.); carrier gas, helium at 1 mL/min; oven temperature, 35 °C maintained for 5 min, increased to 140 °C at a rate of 4 °C/min and maintained for 5 min at 140 °C, increased further to 250 °C at a rate of 10 °C/min, with a final 5 min extension at 250 °C; ion source temperature, 230 °C; transfer line temperature, 250 °C; scan range, 40~400 *m*/*z*; electronic impact (EI) mode, 70 eV.

Mass spectra in the National Institute of Standards Technology Mass Spectral Database 11 (accessed using software from Agilent Technologies, Inc.) were used to identify all of the volatile flavour compounds. The MassHunter workstation was used to compare the MS peaks. The concentration of each compound was calculated using the following formula:

(3) $$c_{i}=\frac{A_{i}}{A_{s}}\times c_{s}$$

where ‘c_i_’ is the concentration (µg/L) of each volatile flavour compound, ‘c_s_’ is the concentration (µg/L) of the ISTD, ‘A_i_’ is the chromatographic peak area of each compound, and ‘A_s_’ is the chromatographic peak area of the ISTD.

The retention index (RI) was calculated from the retention time by automatically retrieving the mass spectral results of each component using the random carry MassHunter workstation Standards Technology Mass Spectral Database 11. RI was calculated using the following formula:

(4) $$\mathrm{RI}=100\times \left[Z+\frac{{\mathrm{RT}}_{\left(X\right)}-{\mathrm{RT}}_{\left(Z\right)}}{{\mathrm{RT}}_{\left(Z+1\right)}-{\mathrm{RT}}_{\left(Z\right)}}\right]$$

where ‘RT’ is the retention time (min); retention times for n-alkane carbon atom numbers follow the RT_(z)_ < RT_(x)_ < RT_(z + 1)_ order.

### 4.9. Determination of the Odour Activity Value (OAV)

To evaluate the influence of individual volatile flavour compounds on aroma, we calculated the OAV, i.e., the ratio of the concentration of a compound to its detection threshold concentration. Additionally, the thresholds for different volatile flavour compounds in dairy products were determined from the literature [57].

### 4.10. Sensory Analysis

Sensory analysis is an effective tool for ensuring the sensory quality, acceptability and identifying defects in dairy products [58]. The sensory panel was comprised of 15 highly experienced and screened judges who were familiar with dairy products. Before the tasting session, the panellists were forbidden from eating for at least 1 h. Then, they worked individually, evaluated the samples in an independent booth and rinsed their mouths with distilled water before tasting the next sample. Each judge on the sensory panel performed the test three times, with an interval of 24 h between sessions. Colour, taste, aroma and texture of the samples were graded using the 100-point intensity scale according to the Chinese dairy industry guideline RHB 103–2004. The colours of the yogurts were determined using a ten-point scale. Taste and aroma were determined between a range of 0–40, and texture was measured on a 50-point scale.

### 4.11. Statistical Analyses

Statistical analyses were conducted using Excel (Microsoft Corp., Redmond, WA, USA) and Origin (OriginLab, Northampton, MA, USA). All experimental data were analysed using the MetaboAnalyst 3.0 (http://www.metaboanalyst.ca) website for statistical analysis. Model quality of the partial least squares discriminant analysis (PLS-DA) was evaluated with the goodness of fit (R^2^Y) and the predictive ability (Q^2^). Prior to statistical analysis, all raw data were mean-centred and pareto-scaled using Statistical Analysis module in MetaboAnalyst. All experiments were conducted with three biological replicates.

## 5. Conclusions

In this study, the physicochemical characteristics of milk fermented using mixed starter cultures with different proportions of *L. delbrueckii* subsp. *bulgaricus* IMAU20312 and *S. thermophilus* IMAU80809 were characterised and the effects of the volatile flavour of the starter cultures investigated using SPME–GC–MS. One mixed-species starter culture, A3, was screened out. At the end of fermentation, all treatments had a viable cell count of >10 ^7^ CFU/mL and a viscosity of 615 mPa·s. There were significant changes in the profiles of the volatile flavour compounds depending on the ratio of the proportion of *L. delbrueckii* subsp. *bulgaricus* IMAU20312 and *S. thermophilus* IMAU80809. A total of 63 volatile compounds and 10 odour-active compounds were identified in all samples. 2-nonanone and formic acid ethenyl ester had high OAVs and were the characteristic odour-active compounds in the A3 samples. In addition, the heatmap and PCA results showed that the flavour components and concentrations of the A3 samples were closer to the commercial control than the other samples. The results of the sensory evaluation also showed a high degree of agreement with the heatmap and PCA results. These results contribute to a better understanding of the effect of starter cultures on the odour quality of fermented milk. The selection of the appropriate proportions of *L. delbrueckii* subsp. *bulgaricus* and *S. thermophilus* as starter cultures is important in determining the final profile of volatiles and the overall flavour of the milk product.

## Figures and Tables

**Table 1:** Changes in viable cell counts of yogurts produced during fermentation using different ratios of *L. delbrueckii* subsp. *bulgaricus* IMAU20312 and *S. thermophilus* IMAU80809 in starter cultures compared with the commercial starter culture.

No.	Fermentation Time (h)	Viable Cell Counts (×10^7^ CFU/mL)				
		0 h	2 h	4 h	6 h	8
A1	6.5	0.55 ± 0.02 ^a^	0.53 ± 0.01 ^e^	0.68 ± 0.09 ^d^	3.14 ± 0.04 ^c^	5.73 ± 0.01 ^a^
A2	7.2	0.59 ± 0.05 ^a^	0.65 ± 0.03 ^d^	0.86 ± 0.03 ^cb^	3.23 ± 0.35 ^c^	5.75 ± 0.05 ^a^
A3	7.5	0.70 ± 0.06 ^a^	0.96 ± 0.02 ^a^	1.09 ± 0.13 ^a^	3.85 ± 0.13 ^b^	5.68 ± 0.55 ^a^
A4	8.0	0.65 ± 0.35 ^a^	0.71 ± 0.01 ^cd^	0.81 ± 0.04 ^cd^	1.69 ± 0.16 ^d^	5.69 ± 0.76 ^a^
A5	7.4	0.68 ± 0.05 ^a^	0.82 ± 0.03 ^b^	0.9 ± 0.07 ^cb^	4.38 ± 0.16 ^a^	5.59 ± 0.55 ^a^
JD	7.2	0.64 ± 0.32 ^a^	0.72 ± 0.07 ^c^	0.96 ± 0.02 ^ab^	3.76 ± 0.15 ^b^	5.70 ± 0.08 ^a^

Note: Figures in the same column followed by different lowercase letters indicate that they are significantly different from each other (*p* < 0.05). The second column represents the time at which the fermented milk samples reached the end of fermentation.

**Table 2:** Differences in water holding capacity and viscosity of yogurts produced through fermentation using different ratios of *L. delbrueckii* subsp. *bulgaricus* IMAU20312 and *S. thermophilus* IMAU80809 in starter cultures compared with the commercial starter culture.

Time (h)	Viscosity (mPa·s)						Water Holding Capacity (%)					
	A1	A2	A3	A4	A5	JD	A1	A2	A3	A4	A5	JD
0	175 ± 13 ^c^	225 ± 1 ^a^	210 ± 1 ^b^	212 ± 1 ^b^	140 ± 1 ^d^	180 ± 5 ^c^	21 ± 0.5 ^b^	28 ± 3.5 ^a^	15 ± 0 ^c^	14 ± 1.5 ^c^	17 ± 2.5 ^c^	15 ± 0.5 ^c^
2	220 ± 2 ^b^	320 ± 3 ^a^	310 ± 8 ^a^	315 ± 6 ^a^	160 ± 24 ^c^	330 ± 3 ^a^	34 ± 0 ^a^	30 ± 2.5 ^a^	22 ± 1 ^b^	19 ± 4.5 ^b^	22 ± 0.5 ^b^	20 ± 3 ^b^
4	340 ± 12 ^c^	420 ± 2 ^b^	440 ± 3 ^d^	300 ± 1 ^d^	335 ± 16 ^c^	470 ± 3 ^a^	47 ± 0.5 ^a^	45 ± 5 ^a^	47 ± 2.5 ^a^	36 ± 2.5 ^b^	25 ± 0.25 ^d^	30 ± 0.5 ^c^
6	470 ± 28 ^c^	510 ± 7 ^b^	495 ± 10 ^a^	370 ± 1 ^d^	470 ± 13 ^c^	525 ± 1 ^b^	48 ± 2.5 ^a^	48 ± 2.5 ^a^	44 ± 0.5 ^b^	42 ± 3 ^b^	45 ± 1 ^ab^	42 ± 0.5 ^b^
8	580 ± 3 ^b^	590 ± 3 ^a^	615 ± 1 ^d^	560 ± 2 ^d^	580 ± 3 ^c^	590 ± 4 ^b^	49 ± 0.5 ^c^	49 ± 0.5 ^b^	57 ± 0.5 ^a^	43 ± 1 ^d^	46 ± 0.5 ^c^	47 ± 0.2 ^b^

Note: Data are represented as means ± SD; figures in the same row followed by different lowercase letters indicate that they are significantly different from each other (*p* < 0.05).

**Table 3:** Sensory evaluation of yogurts produced through fermentation using different ratios of *L. delbrueckii* subsp. *bulgaricus* IMAU20312 and *S. thermophilus* IMAU80809 in starter cultures compared with the commercial starter culture.

No.	Colour	Taste and Flavour	Texture	Total Score
A1	8.00 ± 0.75 ^a^	29.44 ± 1.47 ^c^	35.75 ± 1.46 ^c^	72.34 ± 2.42 ^d^
A2	8.59 ± 0.54 ^a^	32.56 ± 1.32 ^b^	35.65 ± 1.25 ^c^	75.41 ± 2.22 ^c^
A3	8.74 ± 0.60 ^a^	34.56 ± 1.25 ^ab^	44.32 ± 1.59 ^a^	89.80 ± 1.76 ^a^
A4	8.63 ± 0.71 ^a^	34.12 ± 1.11 ^ab^	40.24 ± 1.68 ^b^	82.80 ± 1.89 ^b^
A5	8.60 ± 0.57 ^a^	33.75 ± 1.65 ^ab^	37.38 ± 1.82 ^c^	77.38 ± 1.80 ^c^
JD	8.92 ± 0.84 ^a^	35.79 ± 1.49 ^a^	42.31 ± 1.64 ^ab^	90.19 ± 1.76 ^a^

Note: Data are represented as mean ± SD; figures in the same column followed by different lowercase letters are significantly different from each other (*p* < 0.05).

**Table 4:** Odour activity values (OAVs) of the volatile flavour compounds produced in yogurts following fermentation with different ratios of *L. delbrueckii* subsp. *bulgaricus* IMAU20312 and *S. thermophilus* IMAU80809 in starter cultures compared with the commercial starter culture.

No.	Volatile Compound	Odour Threshold (µg/L)	OAV						References
			A1	A2	A3	A4	A5	JD	
1	Acetaldehyde	8.7	0.68	-	0.32	2.57	-	-	Ning et al., 2011 [42]
2	Benzaldehyde	24	0.10	0.03	0.005	0.05	0.05	0.01	Liu et al., 2014 [43]
3	3-Methyl-butanal	5.4	0.18	-	-	0.18	0.19	-	Qian et al., 2003 [44]
4	Decanal	3	-	0.09	0.05	0.40	0.24	0.03	Curioni et al., 2002 [45]
5	Nonanal	1	0.21	0.49	0.25	2.02	1.22	0.19	Gemert, 2003 [46]
6	2,3-Butanedione	10	-	0.47	0.24	-	-	0.12	Qian et al., 2003 [44]
7	2-Nonanone	5	1.99	0.60	0.93	1.93	1.73	0.47	Curioni et al., 2002 [45]
8	Acetoin	55	0.08	0.30	0.05	0.39	0.14	0.08	Qian et al., 2003 [44]
9	1-Nonanol	45.5	-	-	-	0.07	0.04	0.001	Curioni et al., 2002 [45]
10	Formic acid ethenyl ester	9	-	-	1.41	-	-	-	Gemert, 2003 [46]

**Table 5:** Five ratios of *L. delbrueckii* subsp. *bulgaricus* IMAU20312 and *S. thermophilus* IMAU80809 that were used as mixed starter cultures in this study.

No.	Different Proportional Combinations
A1	1:1
A2	1:10
A3	1:100
A4	1:1000
A5	1:2000
JD	A commercial yogurt starter culture supplied by Chr-Hansen

## Data Availability

Not applicable.

## Supplementary Materials

The following supporting information can be downloaded at: https://www.mdpi.com/article/10.3390/molecules28052123/s1, Table S1: Volatile compounds produced in yogurts with different ratios of *L. delbrueckii* subsp. *bulgaricus* IMAU20312 and *S. thermophilus* IMAU80809 in starter cultures and in the commercial starter culture.
