# Supplementary material for: Influence of Different Ratios of Lactobacillus delbrueckii subsp. bulgaricus and Streptococcus thermophilus on Fermentation Characteristics of Yogurt
Source: Molecules. 2023 Feb 24;28(5):2123. doi: 10.3390/molecules28052123 (PMC10004190; doi:10.3390/molecules28052123)
Supplement: Supplementary file 1 [file molecules-28-02123-s001.zip › molecules-2182010-supplementary.pdf]

Supplementary Material

Influence of Different Ratios of *Lactobacillus delbrueckii* subsp. *bulgaricus* and *Streptococcus thermophilus* on Fermentation Characteristics of Yogurt

Tong Dan [1,2,3,*], Haimin Hu [1,2,3], Jiale Tian [1,2,3], Binbin He [1,2,3], Jiahui Tai [1,2,3] and Yanyan He [1,2,3]

1 Key Laboratory of Dairy Biotechnology and Engineering, Ministry of Education, Department of Food Science and Engineering, Inner Mongolia Agricultural University, Hohhot 010018, China
2 Key Laboratory of Dairy Products Processing, Ministry of Agriculture and Rural Affairs, Department of Food Science and Engineering, Inner Mongolia Agricultural University, Hohhot 010018, China
3 Inner Mongolia Key Laboratory of Dairy Biotechnology and Engineering, Hohhot 010018, China
* Correspondence: dantong813218@aliyun.com

Table S1. Volatile compounds produced in yogurts following fermentation by different ratios of *L. delbrueckii* subsp. *bulgaricus* IMAU20312 and *S. thermophilus* IMAU80809 in starter cultures compared with the commercial starter culture.

No.	Volatile compound	Chemical formula	RI[1]	RI[2]	RT[3] (min)	µg/L					
						A1	A2	A3	A4	A5	JD
**Carboxylic acids**											
1	Alanine	$C_3H_7NO_2$	-	-	1.132	-	7.57	0.81	-	-	0.19
2	D-Alanine	$C_3H_7NO_2$	-	-	1.098	6.90	-	18.52	-	9.93	4.15
3	DL-Alanine, N-acetyl-	$C_5H_9NO_3$	-	-	1.785	25.29	-	-	25.29	52.11	-
4	Acetic acid	$C_2H_4O_2$	633.84	638	2.71	1.80	0.57	-	-	-	0.17
5	2-Pyridinecarboxylic acid	$C_6H_5NO_2$	765	nf	4.267	-	0.56	-	-	-	0.16
6	Propanedioic acid, propyl-	$C_6H_{10}O_4$	829.33	nf	5.93	0.62	2.65	0.58	1.82	4.89	-
7	Hexanoic acid	$C_6H_{12}O_2$	998.48	999	15.06	2.95	11.32	2.97	12.10	8.54	1.69
8	Heptanoic acid	$C_7H_{14}O_2$	1082	1080	16.387	-	0.19	-	23.83	-	-
9	Cyclohexanecarboxylic acid	$C_7H_{12}O_2$	1130.56	1127.00	17.611	0.095	0.15	-	-	-	0.14
10	Octanoic acid, 7-oxo-	$C_8H_{14}O_3$	1109.03	-	19.894	-	0.28	0.12	2.05	0.16	0.03
11	Octanoic acid	$C_8H_{16}O_2$	1189.21	1189	20.21	2.94	1.33	0.24	12.10	8.53	1.69
12	Nonanoic acid	$C_9H_{18}O_2$	1232	1226	22.138	0.16	0.16	-	0.52	-	0.072
13	n-Decanoic acid	$C_{10}H_{20}O_2$	1381.30	1387	24.779	0.34	0.12	0.36	1.86	1.14	-
14	Dodecanoic acid	$C_{12}H_{24}O_2$	1608	nf	30.109	0.13	0.14	0.09	0.94	0.15	0.03
15	Dodecanoic acid, 3-hydroxy-	$C_{12}H_{24}O_3$	1651	nf	30.123	-	0.46	-	-	-	0.78
16	Tetradecanoic acid	$C_{14}H_{28}O_2$	-	-	33.519	0.08	0.35	0.12	1.58	0.55	0.05
17	n-Hexadecanoic acid	$C_{16}H_{32}O_2$	-	-	35.956	0.14	1.04	0.22	0.36	1.03	0.25
18	Oleic Acid	$C_{18}H_{34}O_2$	-	-	36	-	0.36	-	-	0.19	-
19	Propanoic acid, 2-oxo-	$C_3H_4O_3$	659	nf	3.604	0.49	-	-	-	-	0.16
**Aldehydes**											
1	Acetaldehyde	C_2H_4O	-	-	1.72	5.94	-	2.79	22.33	-	-
2	Butanal, 3-hydroxy-	$C_4H_8O_2$	639	nf	2.424	2.34	0.43	0.40	3.88	5.83	0.11
3	Butanal, 3-methyl-	$C_5H_{10}O$	697.86	689	5.587	0.96	-	-	0.96	1.02	-
4	Heptanal	$C_7H_{14}O$	913.65	907	10.84	1.19	-	1.19	0.79	0.71	0.73
5	Benzaldehyde	C_7H_6O	971.05	971	13.72	2.40	0.61	0.12	1.28	1.30	0.33
6	Nonanal	$C_9H_{18}O$	1118.78	1113	18.63	0.21	0.49	0.25	2.02	1.22	0.19
7	Decanal	$C_{10}H_{20}O$	1221	1219	21.68	-	0.28	0.15	1.21	0.71	0.09
**Ketones**											
1	2-Butanone, 3-methyl-	$C_5H_{10}O$	665	666	1.863	1.38	-	-	25.07	30.07	1.32
2	2,3-Butanedione	$C_4H_6O_2$	-	-	2.51	-	4.72	2.39	-	-	1.22
3	2-Pentanone	$C_5H_{10}O$	684.64	685	3.106	0.39	1.27	0.62	5.98	5.43	0.24
4	2,3-Pentanedione	$C_5H_8O_2$	699	697	3.222	0.55	-	1.79	4.57	2.03	3.52

5	Acetoin	$C_4H_8O_2$	713	712	4.08	4.36	16.59	2.65	21.51	7.86	4.35
6	2-Nonanone	$C_9H_{18}O$	1106.15	1104	17.54	9.94	2.98	4.64	9.65	8.63	2.36
7	2-Undecanone	$C_{11}H_{22}O$	1310.42	1305	20.281	0.89	2.49	0.92	8.58	1.92	0.45
8	2H-Pyran-2-one, tetrahydro-6-pentyl-	$C_{10}H_{18}O_2$	1512.66	1505	22.578	-	1.09	-	4.02	3.26	0.56
9	2H-Pyran-2-one, 6-heptyltetrahydro-	$C_{12}H_{22}O_2$	1054.34	1054	32.812	0.60	1.87	0.13	6.04	0.86	0.04
**Alcohols**											
1	1-Butanol, 3-methyl-	$C_5H_{12}O$	733.79	734	4.789	-	0.57	-	-	0.34	-
2	2-Hexanol, 3-methyl-	$C_7H_{16}O$	905	906	4.808	-	-	-	8.93	-	-
3	2-Pentenal, (E)-	C_5H_8O	766	765	5.388	0.22	0.22	0.14	-	-	-
4	1-Hexanol	$C_6H_{14}O$	881.82	880	9.65	0.17	0.49	0.36	2.05	2.34	0.26
5	1-Heptanol	$C_7H_{16}O$	983.46	974	13.43	0.34	1.04	0.64	4.06	3.94	-
6	1-Nonanol	$C_9H_{20}O$	1167.87	1171	19.265	-	-	-	3.25	1.97	0.06
7	Cyclobutanol	C_4H_8O	1181.18	nf	19.83	-	-	3.78	-	-	-
8	2-Nonanol	$C_9H_{20}O$	1107	1108.7	17.56	0.82	-	-	-	-	-
9	1-Octanol, 2-butyl-	$C_{12}H_{26}O$	1279.00	1277(DB-5)	28.546	-	0.11	0.09	1.31	-	0.05
**Esters**											
1	Formic acid, ethenyl ester	$C_3H_4O_2$	-	-	2.45	-	-	12.66	-	-	-
2	Hexanoic acid, ethyl ester	$C_8H_{16}O_2$	1013.73	1014	9.56	0.09	0.20	0.14	0.67	0.69	0.09
3	Octanoic acid, ethyl ester	$C_{10}H_{20}O_2$	-	-	10.16	-	-	0.22	-	-	0.55
4	Acetic acid, 3,7,11,15-tetramethyl-hexadecyl ester	$C_{22}H_{44}O_2$	-	-	33.896	0.64	0.64	-	1.90	0.42	-
**Aromatic hydrocarbons**											
1	Ethylene oxide	C_2H_4O	-	-	1.103	-	8.20	-	-	-	0.72
2	1,3,5-Cycloheptatriene	C_7H_8	761	765	4.639	0.17	0.39	0.46	0.38	1.92	-
3	Butane, 1-chloro-3-methyl-	$C_5H_{11}Cl$	754	nf	4.716	0.10	-	0.33	-	3.03	0.20
4	o-Xylene	C_8H_{10}	901	902	8.566	0.19	0.19	0.14	0.21	-	0.04
5	1,3,5,7-Cyclooctatetraene	C_8H_8	752	758	9.393	-	0.49	0.34	-	0.39	0.11
6	Oxime-, methoxy-phenyl-	$C_8H_9NO_2$	926.73	-	10.753	0.33	0.40	0.10	1.99	3.62	2.40
7	(+)-4-Carene	$C_{10}H_{16}$	944.21	943	11.043	-	0.20	0.07	1.36	-	0.14
8	Pentane, 1-chloro-	$C_5H_{11}Cl$	751	nf	4.803	-	0.29	-	0.98	-	-
9	p-Xylene	C_8H_{10}	878.32	876	8.494	0.12	0.24	-	-	-	-
10	1-Nonyne	C_9H_{16}	1139	nf	16.087	0.27	0.79	0.45	2.61	0.60	0.30
11	Tetradecane	$C_{14}H_{30}$	1439.74	nf	25.644	0.06	0.04	0.09	1.30	0.13	0.03

12	Octadecane, 6-methyl-	$C_{19}H_{40}$	1739.25	1842 (Squalane)	28.546	0.16	0.16	-	-	-	0.12
13	Pentadecane	$C_{15}H_{32}$	1296.47	-	28.537	-	-	0.13	0.26	-	-
14	Octane, 2,7-dimethyl-	$C_{10}H_{22}$	28.546	933.18	9.34	0.09	-	-	-	-	0.15
15	2-Propanamine	C_3H_9N	-	-	1.56	-	6.95	-	31.58	-	2.14
16	Oxetane, 2-methyl-4-propyl-	$C_7H_{14}O$	-	-	19.9	-	-	-	0.18	-	-
15	Butylated Hydroxytolue-ne	$C_{15}H_{24}O$	1566.61	1533.3	28.958	-	-	0.05	1.52	0.15	-

[1]RI, retention index. The RI of unknown compounds calculated against the GC-MS retention time of n-alkanes (C3-C25); [2]RI from a database (http://webbook.nist.gov/chemistry); [3]RT, retention time; nf, the corresponding reference retention index is not found in this database; '-', not detected. Five different ratios of *L. delbrueckii* subsp. *bulgaricus* IMAU20312 and *S. thermophilus* IMAU80809 were used as mixed-starter cultures: A1 =1:1, A2 = 1:10, A3=1:100, A4=1:1000, A5=1:2000, A = JD (commercial yogurt starter culture as a control).
